# Stress and Epilepsy: Towards Understanding of Neurobiological Mechanisms for Better Management

**DOI:** 10.1523/ENEURO.0200-23.2023

**Published:** 2023-11-02

**Authors:** Dhanisha J. Jhaveri, Aileen McGonigal, Christel Becker, Jean-Jacques Benoliel, L. Sanjay Nandam, Lisa Soncin, Iliana Kotwas, Christophe Bernard, Fabrice Bartolomei

**Affiliations:** 1 Queensland Brain Institute, The University of Queensland, Brisbane, QLD 4067, Australia; 2 Mater Research Institute, Faculty of Medicine, The University of Queensland, Brisbane, QLD 4067, Australia; 3 Mater Epilepsy Unit, Department of Neurosciences, Mater Hospital, Brisbane, QLD 4101, Australia; 4 Institut National de la Santé et de la Recherche Médicale, Unité 1124, Université Paris Cité, Paris, 75006, France; 5 Site Pitié-Salpêtrière, Service de Biochimie Endocrinienne et Oncologie, Assistance Publique Hôpitaux de Paris, Sorbonne Université, Paris, 75651, France; 6 Turner Inst for Brain & Mental Health, Faculty of Medicine, Nursing and Health Sciences, School of Psychological Sciences, Monash University, Melbourne, 3800, Australia; 7 Institut National de la Santé et de la Recherche Médicale, Institut de Neurosciences des Systèmes, Aix Marseille University, Marseille, 13005, France; 8 Laboratoire d’Anthropologie et de Psychologie Cliniques, Cognitives et Sociales, Côte d'Azur University, Nice, 06300, France; 9 Epileptology and Cerebral Rhythmology, Assistance Publique Hôpitaux de Marseille, Timone Hospital, Marseille, 13005, France

**Keywords:** anxiety, depression, epilepsy, epileptogenesis, neuroplasticity, stress

## Abstract

Stress has been identified as a major contributor to human disease and is postulated to play a substantial role in epileptogenesis. In a significant proportion of individuals with epilepsy, sensitivity to stressful events contributes to dynamic symptomatic burden, notably seizure occurrence and frequency, and presence and severity of psychiatric comorbidities [anxiety, depression, posttraumatic stress disorder (PTSD)]. Here, we review this complex relationship between stress and epilepsy using clinical data and highlight key neurobiological mechanisms including the hypothalamic-pituitary-adrenal (HPA) axis dysfunction, altered neuroplasticity within limbic system structures, and alterations in neurochemical pathways such as brain-derived neurotrophic factor (BNDF) linking epilepsy and stress. We discuss current clinical management approaches of stress that help optimize seizure control and prevention, as well as psychiatric comorbidities associated with epilepsy. We propose that various shared mechanisms of stress and epilepsy present multiple avenues for the development of new symptomatic and preventative treatments, including disease modifying therapies aimed at reducing epileptogenesis. This would require close collaborations between clinicians and basic scientists to integrate data across multiple scales, from genetics to systems biology, from clinical observations to fundamental mechanistic insights. In future, advances in machine learning approaches and neuromodulation strategies will enable personalized and targeted interventions to manage and ultimately treat stress-related epileptogenesis.

## Significance Statement

Stress contributes to epileptogenesis, to seizure occurrence and to occurrence of psychiatric comorbidities such as anxiety and depression. In this review, we discuss current knowledge of both clinical aspects and neurobiological mechanisms of epilepsy and stress, and identify avenues for further research that could help reduce or prevent epileptogenesis.

## Introduction

Epilepsy is the commonest severe chronic neurologic condition, characterized by the tendency to have recurrent spontaneous seizures caused by transient abnormalities of brain electrical activity, affecting around 50 million people worldwide. However, seizures represent only part of the burden of disease, as psychiatric comorbidities such as depression and anxiety in people with epilepsy are up to eight times more common compared with the general population, and are a critical contributor to overall disability (Keezer et al., 2016). Epidemiological data suggest a bidirectional link between epilepsy and psychiatric comorbidities, with some shared pathogenic mechanisms that remain to be elucidated (Hesdorffer et al., 2012). Because of its impact on public health, epilepsy is the focus of a current World Health Organization Intersectoral Global Action Plan on brain health, with improved service provision for epilepsy including psychosocial care highlighted as a key strategic objective over the next 10 years (The Lancet N, 2022).

Stress has been identified as a major contributor to human disease (Cohen et al., 2007), and a role of stress has been postulated in both the underlying pathophysiological disease process (epileptogenesis; Becker et al., 2015) and in disease burden in terms of symptomatic load. The pathologic effects of chronic stress appear to be determined both by background “resilience” or “diathesis” (vulnerability) and the timing of stressor(s) during the lifespan (Monroe and Simons, 1991; Lupien et al., 2009; Kumar et al., 2011). In particular, the effects of stress on epilepsy symptomatic load can be viewed as (1) stress as a trigger for seizures, affecting their frequency and severity; and (2) stress as a risk factor for developing psychiatric comorbidities such as anxiety and depression, which are known to result in poorer quality of life (Cramer et al., 1998).

**Figure 1.**
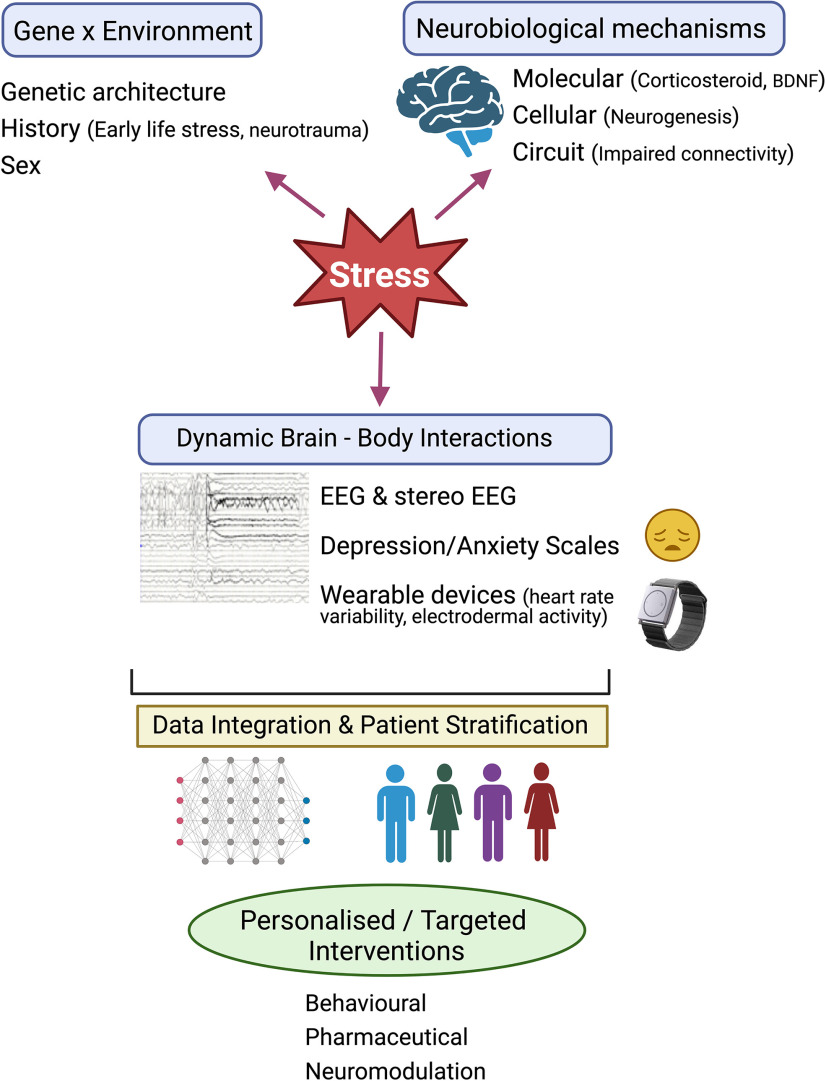
A proposed framework toward personalized and targeted interventions for stress-related epileptogenesis. Stress plays a major role in the pathophysiology of epileptogenesis. Gene x environment interactions including early life stress, neurotrauma underpin this complex relationship between stress and epilepsy leading to impairments in select brain structures and functions. Identifying key neurobiological mechanisms (for example, alterations in neurogenesis, BDNF levels, neural connectivity) that can serve as strong biomarkers together with measurement of brain-body interactions (for example, changes in electrical activity in the brain, electrodermal activity, heart rate variability) could offer a powerful framework to stratify patients with high vulnerability to stress and related psychiatric disorders (depression/anxiety scales). This would require development of new and robust algorithms to integrate data across multiple scales including genetic architecture, neurobiological mechanisms, changes in brain activity and autonomic responses. Use of machine learning and systems neuroscience approaches will guide personalized therapeutic approaches (such as use of behavioral, pharmaceutical, and/or neuromodulatory interventions), to manage stress-related epileptogenesis by refining methods for seizure detection and forecasting and ultimately developing disease-modifying therapies. EEG, electroencephalogram. Created with BioRender.

Here, we review current understanding of the complex relationship between stress and epilepsy and discuss shared molecular and cellular mechanisms based on clinical literature and preclinical research. We note that this is not an exhaustive review of this relationship (for previous review, see Joëls, 2009; Jones and O’Brien, 2013; Jones et al., 2014; Mazarati and Sankar, 2016; Salpekar et al., 2020), but rather a synthesis of knowledge gain by providing examples of clinical and preclinical research conducted in this area. We highlight symptomatic and preventative approaches that could help with manage epilepsy and stress-associated psychiatric conditions and propose a framework for working toward personalized and targeted interventions (Fig. 1).

## Stress and Onset of Epilepsy

Clinical observations of patients whose epilepsy arose following a stressful event, in many cases without additional causes for the epilepsy being identified, were long recognized just as an anecdotal phenomenon (Gastaut and Tassinari, 1966; Temkin and Davis, 1984) with more recent population-based studies providing stronger evidence linking stress and epilepsy onset (Christensen et al., 2007; Shibahara et al., 2013; Y.H. Chen et al., 2017). Epileptic “stress convulsions” were described in the 1970s (Friis and Lund, 1974), with authors hypothesizing that stress reduced the seizure threshold. A retrospective inquiry in a series of patients with epilepsy found that around 5 in 1000 had epilepsy onset in the three months following a major life event such as the death of a close relative, with an average age of onset of around 30 years (Gélisse et al., 2015). Another patient self-report based inquiry revealed differences in stressor type between men and women (Koutsogiannopoulos et al., 2009). This difference has been found in patients with temporal lobe epilepsy (TLE) expressing stress sensitivity, with onset of their epilepsy often being reported as following a psychotraumatic event (Lanteaume et al., 2009). More recently, population studies have reported a stronger evidence of a potential causal link between stress and epilepsy onset: for example, a study from Japan showed an increase in the number of patients with seizures following a life-threatening major natural disaster (earthquake and tsunami in 2011; Shibahara et al., 2013). Another population study from Denmark based on hospital registry data showed that parents who had lost a child had a higher risk of being subsequently diagnosed with epilepsy (Christensen et al., 2007).

Furthermore, several studies have shown a relationship between posttraumatic stress disorder (PTSD) and the development of epilepsy (Zeber et al., 2007; Kessler et al., 2012), for which the term “psychoepileptogenesis” has been proposed (Lanteaume et al., 2009; Soncin et al., 2021). For example, a comprehensive national longitudinal study in Taiwan of individuals with PTSD showed not only an increased risk of developing epilepsy but also earlier age of onset (Y.H. Chen et al., 2017). In addition, Soncin and colleagues recently showed that patients with refractory epilepsy reported more exposure to traumatic events (78% vs 52%) and more symptoms of PTSD (26% vs 7%) than a healthy control group (Soncin et al., 2021). There are strong correlations between seizures, anxiety and fear related to psycho-traumatic exposures associated with the time course of epilepsy (Y.H. Chen et al., 2017). Thus, PTSD probably has an underestimated incidence in patients with epilepsy and remains relatively unstudied.

## Stress as a Trigger for Seizures

Stress is commonly reported as a trigger for seizures in people with epilepsy (C.W. Lai and Trimble, 1997; Galtrey et al., 2016; Kotwas et al., 2017). While a seizure-provoking effect of specific emotion is considered rare (Gastaut and Tassinari, 1966), stress is identified as the most frequent patient-perceived triggering factor (Novakova et al., 2013). Several authors have noted the inherent methodological difficulties in studying the stress-seizure relationship, related to difficulties in quantifying stress and tendency to reporting bias (C.W. Lai and Trimble, 1997; Novakova et al., 2013; Galtrey et al., 2016). Perceived triggering of seizures by stress has been particularly noted in patients with TLE, possibly reflecting a mechanistic role for limbic system dysfunction that alters seizure threshold (Reddy et al., 2021), although systematic studies are lacking. In patients with TLE, differences in brain metabolic activity using 18-flurorodeoxyglucose positron emission tomography (FDG-PET) were observed between those with and without vulnerability to stress/emotional triggers for seizures; vulnerable individuals had more marked anterior temporal lobe hypometabolism including limbic structures (Lanteaume et al., 2012).

## Stress-Related Psychiatric Disorders in Epilepsy: A Bidirectional Link

Chronic stress is considered a key mechanism linking epilepsy and psychiatric disorders such as depression and anxiety as it is a major risk factor for each condition independently (Wulsin et al., 2016). Depression and/or anxiety symptoms are very common epilepsy comorbidities, with prevalence of up to 40% across studies (a higher prevalence than for other chronic medical conditions (such as asthma or diabetes; Lin et al., 2012). These symptoms have a major impact on poorer quality of life in people with epilepsy that is relatively greater than seizure-related factors (E.K. Johnson et al., 2004). This predilection has been attributed to likely effects on brain networks (Colmers and Maguire, 2020), with interactions between epilepsy and psychiatric dysfunction that are bidirectional (Kanner, 2013) and that persist after controlling for common secondary causes of either condition (Keezer et al., 2016). In people with epilepsy, the relationship between seizures and mood-related symptoms can be complex (Colmers and Maguire, 2020); psychiatric symptoms can manifest as interictal or peri-ictal phenomena and can also result as a side effect of treatment (Kanner, 2016). Epilepsy and psychiatric comorbidities can be considered as emergent properties of reorganized brain circuits in the context of a network theory of both epilepsy and mental symptoms (Colmers and Maguire, 2020; Micoulaud-Franchi et al., 2023). Since depression/anxiety in epilepsy can arise either before or after the onset of seizures indicating a bidirectional link, theories of shared pathogenic mechanisms that simultaneously elevate seizure risk and result in mood impairments have been proposed (Bølling-Ladegaard et al., 2023). However, the cellular and molecular mediators underpinning this comorbidity are not clearly understood and remains an active area of investigation.

## Neurobiological Mechanisms Linking Stress, Epilepsy, Depression, and Anxiety

The etiologic link between development of epilepsy and exposure to acute and/or chronic stress has been postulated to reflect the vulnerability of limbic system, in particular the hippocampus and the amygdala to epileptogenesis. Brain regions within the limbic system have differential vulnerability across specific time windows (Lupien et al., 2009), with stress exposure having different effects during the prenatal, early life or adult periods (Gunn and Baram, 2017; Davis et al., 2022). TLE is attributed to abnormal firing in the hippocampus, a key brain area involved in both sensing and regulating the response to stress (McEwen, 1999). Excitation-inhibition imbalance within the hippocampus is one of the shared features of TLE and depression (Danzer, 2012). The amygdala, which plays a key role in emotional circuitry, is also involved in TLE, and amygdalar pathology in patients with epilepsy has been linked to depression and anxiety scores (Tarrada et al., 2022), in terms of connectivity (Doucet et al., 2013) and enlargement (Makhalova et al., 2022). If spontaneous seizures change circuit properties, the latter may further favor occurrence of mental symptoms such as anxiety or depression (Hovatta et al., 2010). Conversely, anxiety may lead to molecular changes, altering neuronal excitability, which may in turn decrease seizure threshold (Borowicz-Reutt and Czuczwar, 2020).

The genetic background and life experiences (and their biological consequences) are unique to individuals (Fig. 1). Using the diathesis-epilepsy framework (Bernard, 2016), we can propose that unresolved stressful experiences may increase the allostatic load and thus diathesis, not only bringing individuals closer to the threshold for seizures and comorbidities, but also possibly lowering the thresholds themselves. Since the way individuals respond to stress depends on their genetic background and their history, which may have left some epigenetic marks, an individual approach is necessary (Bernard, 2016; Ebner and Singewald, 2017). This is relevant given the increasingly recognized diverse array of genetic mechanisms and neurobiological pathways that contribute to epilepsy (Ellis et al., 2020), with complex interactions between genetic and environmental factors that yet to be fully elucidated.

From preclinical studies and limited clinical observations, major mechanisms known to link stress and epileptogenesis include (1) hypothalamic-pituitary-adrenal (HPA) axis dysfunction; (2) altered neuroplasticity within limbic system structures; and (3) specific neurochemical pathways such as brain-derived neurotrophic factor (BNDF), discussed below.

### Hypothalamic-pituitary-adrenal axis dysfunction

Stress, mediated via the hypothalamic-pituitary-adrenal gland (HPA) axis, has been put forward as a candidate mechanism linking epilepsy and psychiatric disorders because of its ability to cause either condition independently (Wulsin et al., 2016). Stress modulates cortisol reactivity via the HPA axis, and assaying cortisol in response to stress exposure has been examined in psychiatric and epileptic populations. There is extensive evidence that stress exposure is associated with abnormal cortisol reactivity across a diverse range of psychiatric disorders (Zorn et al., 2017). Association between stress, seizures and altered cortisol response has also been demonstrated in epileptic populations (van Campen et al., 2016). Repeated early life stress (ELS) produces enduring changes in stress-induced cortisol reactivity that persists into adulthood (Heim et al., 2001). ELS is associated with trauma-related psychiatric disorders (Heim et al., 2001) and in epileptic populations, those who report that stress triggers their seizures, frequently also have a history of ELS (Gulyaeva, 2021).

Chronic abnormalities of stress-related cortisol response in those with ELS might be because of brain region-specific alterations in cellular properties, synaptic connections and dysfunctional functional connectivity that may make an individual more vulnerable to the onset of seizures (Joëls, 2009). For example, 2 h poststress exposure, enhanced connectivity between the amygdala and hippocampus remains persistent (Vaisvaser et al., 2013). In TLE, similar resting state changes have been observed in the hippocampus and amygdala (Allendorfer and Szaflarski, 2014), and may explain the abnormal cortisol response to stress challenge in this population (Allendorfer and Szaflarski, 2014). Changes in resting state functional connectivity (Belleau et al., 2022) and cortisol response to stress challenge are also seen in those with major depression (Morris et al., 2017).

Volumetric loss in brain areas implicated in the HPA axis regulation has been demonstrated via meta-analysis of imaging studies in both epileptic and psychiatric populations (Whelan et al., 2018; Opel et al., 2020). Compared with controls, those with epilepsy show reduced hippocampal and thalamic volume and increased volume of the lateral ventricles (Whelan et al., 2018). Similar findings are seen in a wide range of psychiatric disorders, including major depression and anxiety disorders (Opel et al., 2020). Amygdala enlargement, which is observed in a variable proportion of patients with various form of TLE, has been recently postulated to be associated with stress exposure (Makhalova et al., 2022) but the exact relationship between stress, anxiety and depression has to be confirmed in larger studies. Cortisol hypersecretion decreases seizure threshold, potentially explaining how acute stress can cause seizures in TLE (Feldman and Paul, 1976). The cycle of stress precipitating both psychiatric disorder and seizures dysregulates cortisol activity, leading to a worsening of both conditions.

### Adult hippocampal neurogenesis and neuroplasticity

Both TLE and depression display changes in excitation and inhibition in the hippocampus (Danzer, 2012). How the various hippocampal subfields (specific ones or their combination) contribute to epilepsy and stress is not known. It is likely that multiple possibilities exist, as the answer may be patient specific and time dependent in individuals (Karoly et al., 2021). Experiments focusing on the dentate gyrus of the hippocampus illustrate the current line of reasoning. The dentate gyrus is characterized by a lifelong production and integration of new neurons, granule cells (Ming and Song, 2011; Miller and Sahay, 2019). Impairment in adult neurogenesis has been proposed as an important contributor to epileptogenesis (Jessberger and Parent, 2015). In patients with mesial TLE, a significant decline in neurogenesis and altered gliogenesis correlates with epilepsy duration (Ammothumkandy et al., 2022). Animal models have provided an unprecedented access into the neurogenic changes caused by seizures (Danzer, 2012). In pharmacologically induced seizure models (kainic acid or pilocarpine), substantial disruptions in adult hippocampal neurogenesis have been reported (Parent et al., 1997; Gray and Sundstrom, 1998; Jessberger and Parent, 2015). Following an initial surge in proliferating cells, studies have revealed several maladaptive changes (for review, see Danzer, 2012). These include (1) mossy fiber sprouting with the establishment of unusual synaptic connections onto cells of the inner molecular layer of dentate gyrus and in the CA3 region; (2) abnormal migration of newly generated neurons to ectopic location leading to aberrant integration into the circuit; (3) atypical developmental of basal dendrites on newborn neurons. Such aberrant integration of newly generated neurons in the hippocampus is proposed to contribute to the hyperexcitable circuit observed in TLE. Supporting this notion, new neurons during their immature stages exhibit high vulnerability to seizure-induced abnormal development and alterations in their firing properties (Scharfman et al., 2000; Kron et al., 2010). Interestingly, silencing the activity of adult-born neurons reduces the number of spontaneous recurrent seizures in rodents (Zhou et al., 2019; Lybrand et al., 2021). Thus, impairments in structural connectivity and physiological properties of immature adult-born neurons may contribute to seizure genesis, and perhaps to stress-induced neuropsychiatric conditions such as anxiety and depression. This set of data shows the complexity of the reorganization that can take place in the dentate gyrus. The same amount of detail should be obtained in other hippocampal subfields as well as in connected regions, including the subiculum and the piriform cortex, which are considered as primary epileptogenic zones in TLE (Ben-Ari, 1985).

### Brain-derived neurotrophic factor (BDNF)

Brain-derived neurotrophic factor (BDNF) is a neurotrophin involved in nerve growth, synaptic plasticity (Lu and Gottschalk, 2000), and redox homeostasis (Bouvier et al., 2017). Clinical studies report a tendency to low serum BDNF levels in patients with epilepsy (LaFrance et al., 2010; N.C. Chen et al., 2016; Shpak et al., 2021) or no difference (Hong et al., 2014; Poniatowski et al., 2021). Some studies reported an inverse correlation between seizure frequency and epilepsy duration with serum BDNF levels (Hong et al., 2014; N.C. Chen et al., 2016), including an inverse relation between epilepsy duration and BDNF levels in TLE (N.C. Chen et al., 2016). A relation between BDNF levels and severe forms of epilepsy has been proposed (Poniatowski et al., 2021): BDNF levels could be a biomarker of the worsening epilepsy rather than a biomarker of epilepsy itself. Besides, a cutoff of BDNF level at 6260 pg/ml could predict patients with higher seizure frequency with a sensitivity of 80% and a specificity of 90% (Hong et al., 2014).

Extending this concept to epileptogenesis (the period between the initial insult and the occurrence of the first spontaneous seizures), it can be hypothesized that lower BDNF levels during epileptogenesis could predict epilepsy development. This hypothesis is particularly relevant in the context of past stressful experiences. Intensely stressful experiences produce a decrease in serum BDNF levels (Blugeot et al., 2011). If the stress is not resolved and the allostatic load remains high, conditions may be met to favor epileptogenesis and the expression of comorbidities. In such a context, BDNF levels could be a proxy for diathesis. This scheme has been validated in experimental models. After an intense stressful experience (social defeat) rats split into two groups: those recovering their initial levels of serum BDNF levels and those maintaining stress-induced low levels of serum BDNF (Becker et al., 2015). The latter population develops a depression-like phenotype after being exposed to mild stressful events; they are called vulnerable. The former does not develop a depression-like phenotype; they are called nonvulnerable. This result further supports the idea that rats, even from the same litter, are not homogeneous: they are biologically different. If epileptogenesis is triggered instead of mild stressful events, all rats will develop epilepsy, but the form in vulnerable animals will be more severe and associated with depression-like behavior and cognitive deficits (Becker et al., 2015, 2019). Since low BDNF levels correlate with oxidative stress, treating vulnerable animals with antioxidants during epileptogenesis prevented the development of comorbidities (Becker et al., 2019). Larger prospective clinical studies could be of interest, controlling for treatment, to further investigate the possible role of BDNF as a potential biomarker of epilepsy severity and psychiatric comorbidities.

## Therapeutic Strategies: Symptomatic and Preventative Approaches

### Current clinical management approaches

Management of stress can help optimize seizure control and prevention, as well as psychiatric comorbidities associated with epilepsy. The consequences of psychiatric disorders and epilepsy are inherently stressful events that erode psychosocial resilience (Rutter, 1985; Tedrus et al., 2020), leaving those affected vulnerable to further dysregulated cortisol stress responses. Functional networks modulated by stress appear to be open to therapeutic intervention. For example, vagal nerve stimulation, which is used to treat both epilepsy and depression, causes decreased resting state activation in the hippocampus, amygdala and other regions associated with the HPA axis regulation (Kraus et al., 2007). The current pharmacological treatment options for epilepsy and psychiatric comorbidities are limited and remains a challenge as anti-seizure medications potentially aggravate mood disturbances (Shneker et al., 2009) and antidepressants can lower seizure threshold (Hill et al., 2015).

Various methods, including behavioral, cognitive, and emotional approaches have also been explored to help patients to develop effective strategies to manage stress and seizures (Kotwas et al., 2017). These approaches include cognitive behavioral therapy (CBT) as well as mind-body approaches like mindfulness, meditation, relaxation, and yoga, which target stress management to limit seizure onset and/or their frequency and severity, or seizure control using electrodermal activity biofeedback (Micoulaud-Franchi et al., 2014). Studies have shown that practicing mindfulness may reduce anxiety (Tang et al., 2015), depressive symptoms, and improve quality of life (S.T. Lai et al., 2021), and self-esteem (Dewhurst et al., 2015) in patients with epilepsy. Yoga practice has also shown positive effects on quality of life and seizure frequency (Lundgren et al., 2008). To manage acute stress involved in seizure triggering, electrodermal activity biofeedback has shown some effect on depression and anxiety symptoms. The majority of studies of skin conductance biofeedback in epilepsy have aimed at enhancing tonic levels of sympathetic peripheral system arousal, to reduce cortical excitability and thus increase the seizure trigger threshold; however, the exact mechanisms underlying its therapeutic effect remain to be further investigated (Micoulaud-Franchi et al., 2015).

Although these strategies appear to have a positive effect on patients’ well being (Micoulaud-Franchi et al., 2014; Tang et al., 2014), objective evaluation of precise effects on patients’ physical and mental states by attentional training can be challenging. Whilst these approaches cannot replace drug treatments, they remain attractive adjunct interventions that help patients’ quality of life (Kotwas et al., 2016). Notably, most studies to date have involved small populations of patients with epilepsy, in which distinguishing intervention-related therapeutic effect from nonspecific placebo effect is a major challenge. Only a few studies have attempted to combine observations of clinical change with data from imaging, electrophysiology, or biological markers. One example is a controlled study of electrodermal biofeedback performed by Nagai and colleagues, studying 40 patients with drug-resistant TLE (Nagai et al., 2018). This study showed a significant reduction in seizure frequency in patients undergoing biofeedback sessions compared with those who had only usual treatment. In addition, structural and functional MRI analysis revealed that posttherapy seizure reduction was linearly correlated with enhanced functional connectivity between right amygdala and both the orbitofrontal cortex and frontal pole. These clinical and neuroimaging observations suggest a potential mechanism through autonomic biofeedback that may lead to a progressive effect on frontolimbic neurocircuitry, influencing both the regulation of internal bodily arousal and modulating seizure threshold within connected mesial temporal centers. Further studies employing objective measures of nervous system change in conjunction with clinical measures of symptom burden are to be encouraged, to build a more solid base of mechanistic evidence. This could allow better stratification of patient subgroups, for example identifying subjects more likely to benefit from specific therapies which is a current obstacle to providing a personalized therapeutic framework (Fig. 1).

### Future approaches: towards personalized interventions for stress-related epileptogenesis

At present, strategies to prevent epileptogenesis are lacking, and their development would represent a paradigm shift in treatment of epilepsy and associated psychiatric comorbidities (Terrone et al., 2016). Current concepts of epileptogenesis recognize that epileptogenesis is characterized by a continuum of modifications (rather than being a stepwise process; Terrone et al., 2016) and that it includes not only the prodromal “preepileptic” phase preceding the first seizures, but also disease evolution after the onset of seizures (Pitkänen and Engel, 2014). This latter point is important, given that identifying patients at risk of epilepsy before the first seizure is not currently feasible. On the other hand, disease-modifying treatments initiated after the onset of seizures in selected individuals (ideally those with the highest risk of developing severe, drug-resistant epilepsy) could still be effective in reducing the burden of seizures and comorbidities, in reducing mortality risk (including sudden unexpected death in epilepsy and suicide) and in improving quality of life. Clinical studies coupled with anatomic, physiologic and/or biological data will play an important role not only in driving new hypotheses for putative neurobiological mechanisms but also in enabling the development of novel therapeutic strategies. For example, increasing use of wearable devices in the clinical setting would help build large datasets that combine symptom and physiologic data (K.T. Johnson and Picard, 2020), which together with machine learning approaches offer a promising major step toward the development of personalized interventions. Valuable data could also come from stereoelectroencephalography (SEEG), in which multiple cerebral structures are sampled with depth electrodes in some patients undergoing presurgical evaluation. This allows not only study of anatomic correlations of seizures but also records the resting state interictal activity between seizures with millisecond temporal resolution, and is well suited to signal analysis approaches including study of connectivity (Bartolomei et al., 2017). Abnormal interictal activities such as spikes and high frequency oscillations are a hallmark of epileptogenic structures; disturbances of resting state brain activity, such as altered connectivity, may also be related to interictal psychiatric comorbidities. For example, a key role for the amygdala in both interictal and ictal anxiety symptoms has been shown using SEEG (Makhalova et al., 2022; Singh et al., 2022; Tarrada et al., 2022). Future work could aim at combining SEEG data with other noninvasive modalities (e.g., MRI, PET, MEG, autonomic measures using wearable devices) when investigating neural correlates of psychiatric features in patients with epilepsy.

Another emerging approach is the use of long-term ambulatory EEG recordings using implanted subscalp devices, which coupled with heart rate variability data could provide a powerful tool for tracking fluctuations in brain activity and their relationship with symptoms (Duun-Henriksen et al., 2020), and could greatly inform neural correlates of emotion regulation in epilepsy. Such long-term multimodal data are well-suited to be used in conjunction with ecological momentary assessments captured via smartphone application, which allow longitudinal tracking of self-reported mood and mental state fluctuations (Boemo et al., 2022). Neuroscience-based theoretical modeling could also be used to enrich existing epilepsy modelization approaches by integrating phenomenological (i.e., symptom) data, thereby enabling predictive modeling of seizure activity as well as prediction of the evolution of mental symptoms (Micoulaud-Franchi et al., 2023). Such approaches could pave the way not only for refined seizure forecasting but also for the management of mood regulation, which could improve quality of life in epilepsy and also potentially impact mortality risk related to suicidal ideation (Gratch et al., 2021).

A key goal in the development of disease-modifying treatments for epilepsy is to identify noninvasive biomarkers of epileptogenesis, which could facilitate the challenging move from proof-of-concept anti-epileptogenesis studies to validation in preclinical trials and eventually to clinical translation (Terrone et al., 2016). However, identifying suitable biomarkers for clinical use is in itself a huge challenge. For example, observations from preclinical studies indicate the interest of serum BDNF as a promising biomarker for vulnerability to depression associated with epilepsy (Becker et al., 2015); yet, translation to clinical practice is likely to be hampered by the fact that serum BDNF measurement in patients has proven to be heavily influenced by presence of anti-seizure medications (McGonigal et al., 2023). The possibility of recording physiological changes related to stress (such as electrodermal activity and heart rate variability, facilitated by the new generation of wearable devices; Beniczky et al., 2021) in patients with epilepsy, while simultaneously monitoring their brain rhythm correlates could benefit from powerful machine learning approaches to identify phenotypes at high vulnerability to stress. This could be another avenue for investigating potential noninvasive biomarkers and presents an exciting direction toward personalized and targeted interventions (Fig. 1).

In the clinic, there is no universal rule that applies to all patients with epilepsy and instead an individualised approach is necessary. The same concept applies to experimental models. The expression of phenotypes (seizure severity and comorbidities) is both species and strain dependent (Inostroza et al., 2012) and in animal studies, even within the same litter, offspring with epilepsy can express a variety of phenotypes (Medel-Matus et al., 2017), reflecting different genetic backgrounds and life experiences, possibly resulting in different allostatic load and diathesis (Fig. 1). Future experimental studies should include individual biological variability as an independent variable. Meaningful progress will require close collaborations between clinicians and basic scientists to integrate clinical observations and modeling the disease in preclinical studies to obtain fundamental mechanistic insights. Such an approach would include targeting multiple scales, from genetics (M.R. Johnson et al., 2015) to systems biology (Loeb, 2011), in which the various shared mechanisms of stress and epilepsy present multiple possible avenues of research. The validity of this approach is reinforced by the fact that stressful life experiences, while affecting individuals differently, can readily be identified as potential risk factors for developing epilepsy, for seizure occurrence, and for risk and severity of psychiatric comorbidities, and thus present an opportunistic window for investigation and treatments based on understanding neurobiological basis of stress.

## Synthesis

Reviewing Editor: William Stacey, University of Michigan Decisions are customarily a result of the Reviewing Editor and the peer reviewers coming together and discussing their recommendations until a consensus is reached. When revisions are invited, a fact-based synthesis statement explaining their decision and outlining what is needed to prepare a revision will be listed below. The following reviewer(s) agreed to reveal their identity: Nigel Jones.

There are a wide range of topics in the “stress and epilepsy” problem and there have been many reviews over the past 20 years on this subject. Much of this paper is a limited retelling of those past works and is somewhat superficial. Because the topic is so broad, there is a limit to how much can be told. But we agreed that this paper is not a definitive or comprehensive review of the literature. The real key of this work is in the last section (see next comments). However, it is important to admit within the text that this work is not trying to be exhaustive of all these topics, and perhaps more important, to recognize more of the work that has been done in this sphere. Many previous reviews have attempted this over the past 10-12 years, but many of these are not referenced and should at least be respected. Both reviewers thought the choice of attribution was somewhat arbitrary and incomplete. For instance, Dale Hesdorffer from the clinical perspective and Nigel Jones and Terry O’Brien from the discovery side have contributed much that we know about stress and epilepsy but were absent from the review. There are other important examples as well. So we recommend to sharpen up and condense the elements that have been written about many times before and rely more on citing past work and past reviews, rather than making it seem like this is meant to be an introduction to all these subjects. Changing that approach should also serve to tighten up the narrative through much of the paper, which was felt to be sometimes rambling and poorly structured.

One other potential improvement would be to expand possibly on the critical analysis or evaluation of the quality of the various lines of evidence apart from one sentence on page 4 (‘inherent methodological difficulties’). This applies also to the ‘Future approaches’ section, but that section had more novelty in its own right so we treated that section differently.

The novelty of this paper is the final section on ‘Future approaches’, which leads to the figure. We appreciated the novel perspective of examining these interactions from a clinical and personalised treatment perspective, which provides a different angle than previous work. This section should be expanded and sharpened to focus on the novel aspects. In fact, this really should be the main focus of the paper. Rather than having it as a conclusion, the concepts outlined in the future directions and in the Figure should be more successfully integrated into the main text--that should be the main goal of the whole paper. There seems to have been some attempts at this, but since this is the primary angle the authors are taking, it would have been good to see this consistently driven through the paper.

## Author Response

Response to Reviewers

We thank the reviewers for their constructive comments and criticism, which we have addressed below.

1. There are a wide range of topics in the “stress and epilepsy” problem and there have been many reviews over the past 20 years on this subject. Much of this paper is a limited retelling of those past works and is somewhat superficial. Because the topic is so broad, there is a limit to how much can be told. But we agreed that this paper is not a definitive or comprehensive review of the literature. The real key of this work is in the last section (see next comments). However, it is important to admit within the text that this work is not trying to be exhaustive of all these topics, and perhaps more important, to recognize more of the work that has been done in this sphere. Many previous reviews have attempted this over the past 10-12 years, but many of these are not referenced and should at least be respected. Both reviewers thought the choice of attribution was somewhat arbitrary and incomplete. For instance, Dale Hesdorffer from the clinical perspective and Nigel Jones and Terry O’Brien from the discovery side have contributed much that we know about stress and epilepsy but were absent from the review. There are other important examples as well. So we recommend to sharpen up and condense the elements that have been written about many times before and rely more on citing past work and past reviews, rather than making it seem like this is meant to be an introduction to all these subjects. Changing that approach should also serve to tighten up the narrative through much of the paper, which was felt to be sometimes rambling and poorly structured.

We agree with the reviewers that there has been substantial previous literature reviewing the link between stress and epilepsy. To highlight this point, we have now added the following sentence and have cited several previous reviews including those from Dale Hesdorffer, Nigel Jones and Terry O’Brien on page 3.

“We note that this is not an exhaustive review of this relationship, {for previous reviews, see [10-14]} but rather a synthesis of knowledge gain by providing examples of clinical and pre-clinical research conducted in this area.”

Moreover, based on reviewers’ suggestion, we have sharpened and condensed sections and have removed sections titled “Stress and the nervous system” and “Post-traumatic stress disorder (PTSD) and epilepsy: a link to “psychoepileptogenesis” and have re-phrased titles of a few sections (highlighted in red text).

2. One other potential improvement would be to expand possibly on the critical analysis or evaluation of the quality of the various lines of evidence apart from one sentence on page 4 (‘inherent methodological difficulties’). This applies also to the ‘Future approaches’ section, but that section had more novelty in its own right so we treated that section differently.

Where appropriate, we have discussed strengths and limitations of various approaches in our review. However, based on reviewers’ suggestion, we have now added a new paragraph discussing the quality and appropriateness of current approaches and have suggested the use combinatorial approaches in future studies for better stratification of patient subgroups. This is included in the section titled “Current clinical management approaches” on pages 10-11.

3. The novelty of this paper is the final section on ‘Future approaches’, which leads to the figure. We appreciated the novel perspective of examining these interactions from a clinical and personalised treatment perspective, which provides a different angle than previous work. This section should be expanded and sharpened to focus on the novel aspects. In fact, this really should be the main focus of the paper. Rather than having it as a conclusion, the concepts outlined in the future directions and in the Figure should be more successfully integrated into the main text--that should be the main goal of the whole paper. There seems to have been some attempts at this, but since this is the primary angle the authors are taking, it would have been good to see this consistently driven through the paper.

We thank the reviewers for their positive comments related to the section on “Future approaches.” As per their suggestion, we have now substantially expanded this section (new text in red) and have discussed integration of several approaches which are highlighted in the Figure that may aid in refining seizure forecasting and improving the management of mood regulation in patients with epilepsy. We have also integrated and referred to the Figure throughout the main text.
